# Advancing aquafeed: prebiotic, antimicrobial, antioxidant, and immunomodulatory benefits of innovative ingredients from farming systems

**DOI:** 10.3389/fmicb.2026.1764214

**Published:** 2026-02-20

**Authors:** M. Girão, D. Amaral, G. Campos, R. Urbatzka, H. Peres, R. O. A. Ozorio, M. F. Carvalho

**Affiliations:** 1 CIIMAR/CIMAR LA, Interdisciplinary Centre of Marine and Environmental Research, University of Porto, Matosinhos, Portugal; 2 ICBAS–School of Medicine and Biomedical Sciences, University of Porto, Porto, Portugal; 3 Department of Biology, Faculty of Science, University of Porto, Porto, Portugal

**Keywords:** aquaculture, sustainable aquafeed, circular economy, functional feed ingredients, bioactive compounds

## Abstract

Aquaculture plays a vital role in meeting the increasing global food demand driven by population growth. Sustainable development in the sector depends on optimizing fish nutrition and reducing antibiotic use, with bioactive ingredients offering a promising means to enhance animal health and productivity. Applying circular economy principles, specifically valorising underutilized biomasses as functional feed ingredients, provides a synergistic strategy to boost both environmental sustainability and economic efficiency by minimizing waste and input costs. This study investigated waste-derived biomasses from fungi (*Pleurotus ostreatus*), invertebrates (*Tenebrio molitor* and *Eisenia fetida*), microalgae (*Phaeodactylum tricornutum* and *Scenedesmus* spp.) and aquatic plants (*Lemna minor* and *Nasturtium officinale*) sourced from European freshwater aquaculture systems as novel feed ingredients with functional properties. These were screened for their prebiotic potential, as well as for their antimicrobial, antioxidant and immunomodulatory properties. Prebiotic effects were assessed by measuring the ability of the biomasses to promote the growth of seven probiotic bacterial strains relevant to fish and human health. All strains except *Bifidobacterium longum* ATCC15708 showed enhanced growth in the presence of at least one biomass, with strain-specific responses indicating selective prebiotic effects. Organic and aqueous biomass-derived extracts were tested for antimicrobial activity against 13 human and fish pathogens using the disc diffusion assay. Extracts from *P. ostreatus*, *T. molitor*, *E. fetida*, and *L. minor* inhibited growth of *Aeromonas hydrophila*, *A. sobria*, *A. salmonicida*, *Lactococcus garvieae*, *Listonella anguillarum*, *Tenacibaculum maritimum*, and *Staphylococcus aureus*, with inhibition zones ranging from 9–16 mm at 1 mg·mL^−1^ of extract. When tested for their pro- and anti-inflammatory properties in RAW 264.7 cells, several fungal, microalgal, and aquatic plant extracts exhibited significant anti-inflammatory activity by reducing nitric oxide production without affecting cell viability. Antioxidant assessments revealed marked differences among biomasses, with aqueous extracts of aquatic plants showing the highest DPPH radical scavenging capacity, microalgal activity varying with solvent polarity, and fungi and invertebrates exhibiting comparatively low antioxidant potential. Our findings highlight the beneficial properties of low-value, circular-economy biomasses, supporting their development as sustainable, cost-effective aquafeed ingredients. Further research is underway to characterize the *in vivo* effects of these ingredients when incorporated into experimental fish diets.

## 1 Introduction

Aquaculture has become a cornerstone of global food production, playing a vital role in meeting the growing demand for high-quality animal protein. Its rapid expansion, however, is constrained by several challenges, notably the dependence on finite marine resources such as fishmeal and terrestrial crops like soy, which contribute to environmental degradation through overfishing, deforestation, and pollution (Macusi et al., 2023). Furthermore, the vulnerability of aquaculture production systems to disease outbreaks (Cain, 2022) leads to the routine use of antibiotics (Chen et al., 2020), which contributes to the emergence of antimicrobial resistance and poses risks for both environmental health and food safety (Romero et al., 2012). These issues underscore the need for functional, innovative, and sustainably sourced feed alternatives as a critical priority for the sector (Eroldoğan et al., 2023). Bioactive ingredients offering multiple health benefits, such as prebiotic, antimicrobial, antioxidant, and immunomodulatory effects, represent a promising strategy to address this need. By enhancing animal growth performance, supporting beneficial microbiota, and strengthening immune responses, these ingredients can help reduce antibiotic dependency and promote more resilient aquaculture systems, thereby supporting both productivity and sustainability (Iheanacho et al., 2025). Incorporating such ingredients into aquafeeds aligns with the One Health framework and contributes to reducing the ecological footprint of aquaculture (Stentiford et al., 2020). At the same time, applying circular economy principles to aquafeed development provides a compelling model for reconciling sustainability and economic viability. Valorisation of underutilized or by-product biomasses into high-value feed ingredients reduces environmental burdens, minimizes waste, and diversifies raw material supply chains (Kusumowardani and Tjahjono, 2020). Previous studies have highlighted the potential of diverse biomass by-products and side streams from agro-industrial processes as novel feed ingredients (Sandström et al., 2022). However, while the nutritional and functional properties of these biomasses are increasingly recognized, their incorporation into aquafeeds requires consideration of regulatory frameworks and their intrinsic variability. The broader potential of their use can be supported by comprehensive data on their biochemical composition and safety, which are essential for authorization and market uptake. This study aimed to demonstrate *in vitro* the health-promoting potential of by-product-derived biomasses cultivated using nutrient-rich recirculating aquaculture system (RAS) effluents, advancing the knowledge on novel sustainable resources from European freshwater farming systems. All biomasses were produced under these conditions, and included: *Pleurotus ostreatus*, an industrially-relevant species that represents ca. 19% of global mushroom production (Zied and Pardo-Giménez, 2017), reported to have several therapeutic properties including antioxidant, antihyperlipidemic, antitumor, immunoregulatory and bacteriostatic (Zhao et al., 2024); *Tenebrio molitor* and *Eisenia fetida,* two strong insect candidates for fishmeal replacement in aquafeeds due to their high nutritional value (Musyoka et al., 2019; Shafique et al., 2021) and bioactive properties (Bansal et al., 2018; Errico et al., 2022); the microalgae *Phaeodactylum tricornutum* and *Scenedesmus* spp., promising alternative protein and fat sources shown to support fish growth and nutrient utilization while contributing to the reduction of fishmeal dependency (Gong et al., 2019; Sørensen et al., 2016) and displaying relevant biological properties (Ishaq et al., 2016; Khawli et al., 2021); and *Lemna minor* and *Nasturtium officinale*, fast-growing aquatic plants with valuable nutritional (Gong et al., 2019; Sońta et al., 2019) and phytochemical (Ishaq et al., 2016; Seferli et al., 2024) contents that make them suitable candidates for inclusion in aquafeeds. Our findings highlight that these biomasses can serve as cost-effective and sustainable feed ingredients, supporting the transition of aquaculture towards a circular bioeconomy.

## 2 Materials and methods

### 2.1 Biomass processing and chemical extraction

Biomasses from different biological kingdoms – fungi (*Pleurotus ostreatus*), invertebrates (*Tenebrio molitor*, *Eisenia fetida*), microalgae (*Phaeodactylum tricornutum*, *Scenedesmus* spp.), and aquatic plants (*Lemna minor*, *Nasturtium officinale*) (Table 1) – were selected to evaluate their functional properties as potential novel feed ingredients. All biomasses were produced in accordance with circular economy principles, utilizing waste or by-products from other production systems. Briefly, *P. ostreatus* was cultivated using frass from *T. molitor* (TEBRIO, Salamanca, Spain). The frass was mixed at concentrations of 0, 2.5, 5, 7.5, 10, 12.5, and 15% with a commercial mushroom substrate (Micelios Fungisem S. A., Calahorra, Spain), as previously described by Hilali et al. (2025). Both stems and fruiting bodies were used in the experiments. Additionally, mealworm larvae (TEBRIO, Salamanca, Spain), was tested a novel alternative protein source for human and animal nutrition. The redworm (*Eisenia fetida*) was reared in decoupled European perch (*Perca fluviatilis*) aquaponics system (Keywater Fisheries, Sligo, Ireland) utilizing a substrate composed of a mixture of duckweed (*Lemna minor*) and solid aquaponics waste (excreta and uneaten feed). The microalgae *Phaeodactylum tricornutum* and *Scenedesmus* spp. were cultivated independently in a decoupled “algaeponics” system (SYKE, University of Helsinki, Finland), an autotrophic process that utilizes liquid effluent from RAS system of the Finnforel rainbow trout farm (Finland). To ensure culture stability, the effluent was pre-treated via sequential filtration and UVC sterilization to remove native grazers, and it is supplemented with phosphorus and trace metals to correct natural nutrient imbalances. Cultivation is performed semi-continuously in 320 L mesocosms under constant temperature (20 °C), light (120 μmol/m^2^/s) and pH (7.5–8). Fresh samples of duckweed and watercress (*Nasturtium officinale*) were collected directly from the surface of carp (*Cyprinius carpio*) earthen production ponds at Ińskie Centrum Rybactwa carp farm (ICR; Szczecin, Poland). To generate crude extracts for *in vitro* bioactivity assays, all biomass samples were initially subjected to freeze-drying and subsequently ground into a fine powder. A sequential chemical extraction procedure was employed to fractionate compounds based on solvent polarity. For each biomass, 5 g aliquot of the freeze-dried material was subjected to independent extractions with three solvents, applied sequentially in order of decreasing polarity: deionized water, acetone/methanol (1:1, v/v), and dichloromethane. This approach is expected to recover crude extracts enriched in broad classes of solvent-soluble compounds, with aqueous extracts containing predominantly polar constituents (e.g., sugars, peptides, and other polar metabolites), acetone/methanol extracts enriched in semi-polar compounds (e.g., phenolics and pigments), and dichloromethane extracts containing mainly non-polar compounds (e.g., lipophilic metabolites). Extractions were carried out in 100 mL glass flasks by adding 30 mL of the respective solvent, followed by agitation at 200 rpm for 30 min at room temperature (~20 °C). The aqueous or organic layer was collected and the solvent was subsequently removed using a rotary evaporator under reduced pressure. Each extraction was performed in duplicate to improve yield. The extraction efficiency was quantified by determining the weight of the resulting crude extracts relative to the initial biomass weight. Recovered crude extracts were weighed, resuspended in dimethyl sulfoxide (DMSO, ≥99.9%; Sigma-Aldrich, St. Louis, MO, USA), and stored at −20 °C until required further analysis.

**Table 1:** List of biomasses selected for functional evaluation.

Biological group	Species	Common name	Source location
Fungi	*Pleurotus ostreatus*	Oyster mushroom	Mushroom Technological Research Centre of La Rioja, Calahorra, Spain
Invertebrate	*Tenebrio molitor*	Mealworm	TEBRIO, Salamanca, Spain
	*Eisenia fetida*	Redworm	Keywater Fisheries, Sligo, Ireland
Microalgae	*Phaeodactylum tricornutum*	–	Finnish Environment Institute, Helsinki, Finland
	*Scenedesmus* spp.	–	
Aquatic plant	*Lemna minor* (1)*	Duckweed	Keywater Fisheries, Sligo, Ireland
	*Lemna minor* (2)*	Duckweed	Ińsko Fisheries Center, Szczecin, Poland
	*Nasturtium officinale*	Watercress	

*1, Dehydrated; 2, Fresh.

### 2.2 Isolation and taxonomic identification of pathogenic bacterial strains from rainbow trout *Oncorhynchus mykiss*

Aiming to expand our collection to include native fish-pathogenic strains in the *in vitro* antimicrobial screenings, bacterial isolates were obtained from the head kidney, brain, and epidermal mucus of diseased rainbow trout (*Oncorhynchus mykiss*) collected from local freshwater aquaculture facilities. Within 12 h, fish showing external signs of infection were sampled, transported under refrigeration to the laboratory, and processed for bacterial isolation. Five fish were aseptically dissected, and sterile swabs were used to collect samples from each target tissue. Each swab was streaked onto Tryptone Soya Yeast Extract Agar (TSYA; Oxoid, Hampshire, UK) plates, which were incubated aerobically at 28 °C for 96 h (Ortega et al., 2020). All morphologically distinct colonies were re-streaked on fresh agar medium until pure cultures were obtained, which were then preserved at −80 °C in 30% (v/v) glycerol. All isolates were taxonomically identified through sequencing of the 16S rRNA gene. Each isolate was grown in TSYA media to obtain biomass for DNA extraction. Genomic DNA was extracted using the E.Z.N.A. Bacterial DNA Kit (Omega Bio-Tek, GA, United States). The 16S rRNA gene was amplified by PCR using the universal primers 27F/1492R, as described by Girão et al. (2024a). The acquired 16S rRNA gene sequences were analysed using Geneious Prime 2024.0 software (Biomatters, Auckland, New Zealand). The EzTaxon tool from the EzBioCloud database (Yoon et al., 2017) was used to establish strain taxonomic affiliation based on consensus sequence similarity with deposited 16S rRNA data. All generated sequences were deposited in GenBank under the accession numbers PX617744-57(NCBI, Bethesda, MD, United States) (Supplementary Table S1).

### 2.3 *In vitro* bioactivity assays

#### 2.3.1 Prebiotic screening

The prebiotic effect of all biomasses was assessed *in vitro* by evaluating their ability to support and enhance the growth of probiotic bacterial strains relevant to fish and human health. Six reference strains were used – *Bacillus spizizenii* ATCC6633, *Bacillus subtilis* ATCC6051, *Pseudomonas chlororaphis* subsp. *piscium* DSM21509, *Bifidobacterium longum* ATCC15708, *Lactobacillus rhamnosus* ATCC BAA-3227, and *Lactobacillus acidophilus* ATCC314 – along with *Lactococcus raffinolactis* SAFE_10 recovered from diseased *O. mykiss*. This final strain was included due to the described probiotic properties of this species. This panel comprises both Gram-positive and Gram-negative bacteria, including aerobic, facultative anaerobic, and anaerobic/microaerophilic strains, which represent a broad spectrum of phylogenetic and physiological diversity relevant to fish and human health. Each bacterial strain was cultured in Basal Mineral Salts (BMS) medium, composed of 3.0 g/L NaNO₃, 0.5 g/L KCl, 1.0 g/L KH₂PO₄, 0.5 g/L MgSO₄·7H₂O, 0.1 g/L NaCl, and 1 mL/L trace salt solution composed of 1.0 g FeSO₄·7H₂O, 0.1 g CuSO₄·5H₂O, and 0.1 g ZnSO₄·7H₂O dissolved in 100 mL of distilled water, supplemented with 1% (w/v) of each freeze-dried biomass individually. Bacterial growth was assessed under both liquid and solid cultures. All cultures were incubated for 96 h under strain-specific temperature and oxygen conditions (Supplementary Table S2). For *B. longum* ATCC15708, an obligate anaerobic strain, oxygen depletion was achieved using an anaerobic jar system (Thermo Scientific, United States). In liquid media, growth was monitored every 24 h by measuring the culture’s optical density at 600 nm (OD_600_), starting from an initial inoculum standardized to OD_600_ = 0.01. For solid media assays, growth was evaluated qualitatively by visual inspection of colony formation after 96 h of incubation. The growth-promoting effects of biomass supplementation were determined by comparing results with those of the unsupplemented BMS, based on increases in OD_600_ for liquid cultures and the presence of colony development on solid media. The assays were conducted in duplicate.

#### 2.3.2 Antimicrobial screening

The antimicrobial activity of the previously obtained extracts was evaluated using an agar disk diffusion assay, as previously described by Girão et al. (2024b). The extracts were tested against a panel of pathogens relevant to both aquaculture and human health, comprising a diverse range of Gram-positive, Gram-negative, and fungal microorganisms. Aquaculture-relevant strains included *Edwardsiella tarda* DSM30052, *Aeromonas hydrophila* DSM3018, *Pseudomonas anguilliseptica* DSM1211, *Yersinia ruckeri* ATCC29473, *Listonella (Vibrio) anguillarum* ATCC19264, *Tenacibaculum maritimum* ATCC43397, and *Lactococcus garvieae* DSM20684. The human-associated pathogens tested were *Escherichia coli* ATCC25922, *Staphylococcus aureus* ATCC29213, *Salmonella enterica* ATCC25241, and *Candida albicans* ATCC10231. Additionally, two strains isolated from the rainbow trout – *Aeromonas sobria* SAFE_07 and *Aeromonas salmonicida* subsp. *salmonicida* SAFE_15 – were also included in the screening. Each strain was grown in the appropriate culture conditions (Supplementary Table S3). For the assay, a suspension of each strain was prepared in the appropriate liquid medium, with its turbidity adjusted to 0.5 McFarland standard (OD₆₂₅ = 0.08–0.13) (Balouiri et al., 2016). The suspensions were used to evenly seed the surface of agar plates. Blank paper disks (6 mm in diameter) were then placed on the inoculated plates and loaded with 15 μL of each extract (1 mg/mL). Enrofloxacin (1 mg/mL; Sigma-Aldrich, MO, United States) and nystatin (1 mg/mL; Sigma-Aldrich) were used as positive controls for bacterial and fungal strains, respectively. DMSO was used as the negative control. Each extract was tested in two independent replicates. Antimicrobial activity was assessed by measuring the diameter (mm) of the inhibition zones formed around the disks.

#### 2.3.3 Antioxidant screening

The antioxidant activity of the extracts was evaluated using the DPPH (2,2-diphenyl-1-picrylhydrazyl) radical scavenging assay as described by Dulf et al. (2015). Briefly, 40 μL of each extract was mixed with 200 μL of a DPPH solution (0.02 mg/mL). The mixture was incubated at room temperature for 15 min, and the absorbance measured at 517 nm. The assay was performed on a microplate, in duplicate. The extracts obtained with dichloromethane were excluded from the assay due to their poor solubility in methanol, the solvent used for preparing the DPPH reagent.

#### 2.3.4 Immunomodulatory screening

The immunomodulatory properties of the extracts were evaluated *in vitro* using the RAW 264.7 macrophage cell line (American Type Culture Collection, ATCC). Both anti-inflammatory and pro-inflammatory activities were assessed by quantifying nitric oxide (NO) production, as described by Regueiras et al., 2022 (Regueiras et al., 2021), while cytotoxic effects were determined using the MTT [3-(4,5-dimethylthiazol-2-yl)-2,5-diphenyltetrazolium bromide] assay, as described by Girão et al. (2019). RAW 264.7 cells were cultured in Dulbecco’s Modified Eagle Medium (DMEM) (Gibco, Thermo Fisher Scientific, Waltham, MA, United States), supplemented with 10% (v/v) fetal bovine serum (Biochrom, Berlin, Germany), 1% (v/v) antibiotics including 100 mg/L streptomycin, 100 IU/mL penicillin (Biochrom, Berlin, Germany), and 0.1% (v/v) amphotericin B (GE Healthcare, Little Chalfont, UK). Cells were seeded in 96-well plates (3.5 × 10^5^ cells/well) and allowed to adhere overnight. Cultures were maintained at 37 °C in a humidified atmosphere containing 5% CO₂. For the assessment of anti-inflammatory activity, cells in each well were co-exposed to the extracts (15 μg/mL, dissolved in DMSO ≥99.9%.) and lipopolysaccharide (LPS; 1 μg/mL; Sigma-Aldrich, St. Louis, MO, United States). After 24 h of incubation, 75 μL of culture supernatant was transferred to a new 96-well plate and mixed with 75 μL of Griess reagent, composed of 10 mg/mL sulfanilamide and 1 mg/mL N-(1-naphthyl)ethylenediamine dihydrochloride in 2% phosphoric acid, to quantify nitric oxide (NO) production. Following a 10-min incubation in the dark, the absorbance was measured at 562 nm. Results were expressed as the percentage of NO production relative to the LPS-stimulated control (DMSO + LPS). Pro-inflammatory activity was evaluated under the same conditions but without LPS. Results were expressed as the percentage of NO production relative to the solvent control DMSO. The remaining cells from the initial plates, both those used for anti- and pro-inflammatory screening, were used to assess cell viability after extract exposure using the MTT assay. A fresh MTT solution (0.5 mg/mL in DMEM) was prepared, and 100 μL was added to each well. After a 45-min incubation at 37 °C, the medium was carefully removed, and formazan crystals were dissolved in 100 μL of dimethyl sulfoxide (DMSO). Finally, absorbance was measured at 570 nm, and cell viability was expressed as a percentage relative to the solvent control (DMSO). The assays were performed in two independent biological replicates, each conducted in technical triplicate. Data from immunomodulatory assays were tested for significant differences compared to the most appropriate control.

#### 2.3.5 Statistical analysis

All quantitative data are presented as mean ± standard deviation, unless otherwise stated. For immunomodulatory, cytotoxicity and antioxidant assays, differences among treatments were analyzed using one-way analysis of variance (ANOVA) for normally distributed data, followed by Dunnett’s *post hoc* test to compare each treatment with the appropriate control. When data did not meet the assumptions of parametric analysis after transformation, the non-parametric Kruskal–Wallis test was applied, followed by Dunn’s multiple comparison test. A confidence level of 95% was considered in all statistical analyses. The significance level was set at *p* < 0.05 for all tests. All analyses were performed using SPSS (v27, IBM, NewYork, NY, United States).

## 3 Results and discussion

### 3.1 Bacterial strains isolated from *Oncorhynchus mykiss*

A total of 18 bacterial strains were isolated from the epidermal mucus (*n* = 2), head kidney (*n* = 7), and brain (*n* = 9) of diseased *Oncorhynchus mykiss*. These particular tissues were selected to capture key microbial niches relevant to fish health: epidermal mucus as a primary barrier hosting commensal and pathogenic microbes (Dash et al., 2018); head kidney as the central immune organ influencing systemic host–microbe interactions (Geven and Klaren, 2017); and brain to identify neuroinvasive or opportunistic pathogens involved in systemic infections (Neves et al., 2009). This targeted approach enabled the isolation of native pathogenic strains for inclusion in the antimicrobial assays, as well as probiotic strains that were used in the prebiotic assays. The taxonomic identification of these isolates, performed based on 16S rRNA gene similarity with curated databases, revealed representatives affiliated with the genera *Aeromonas*, *Pseudomonas*, *Lactococcus*, *Staphylococcus*, *Acinetobacter*, *Yersinia*, *Iodobacter*, and *Chryseobacterium* (Supplementary Table S1). The majority of these bacteria are Gram-negative, with only *Lactococcus* and *Staphylococcus* being classified as Gram-positive. *Aeromonas* species were isolated from all tissues, which is consistent with their well-documented role as fish pathogens (Dallaire-Dufresne et al., 2014; Wahli et al., 2005). In this context, *A. sobria* SAFE_07 and *A. salmonicida* subsp. *salmonicida* SAFE_15 were selected for our antimicrobial assays, as they represent clinically relevant pathogens in aquaculture (Liu et al., 2025; Park et al., 2020). *L. raffinolactis* strains were detected in both head kidney and brain. Although the presence of these strains in these tissues does not imply a probiotic role, members of this genus are known to include strains with beneficial properties, which justified their inclusion in our prebiotic screening assays (Jung et al., 2020). Other isolates, such as *Pseudomonas* spp. and *Staphylococcus* spp., are known for their diverse ecological roles, including opportunistic pathogenicity and environmental adaptation. Collectively the set of isolates obtained in this study constitutes a resource of biologically and ecologically relevant bacterial strains that may support future functional and pathogenicity studies.

### 3.2 Chemical extraction efficiency

**Figure 1:**
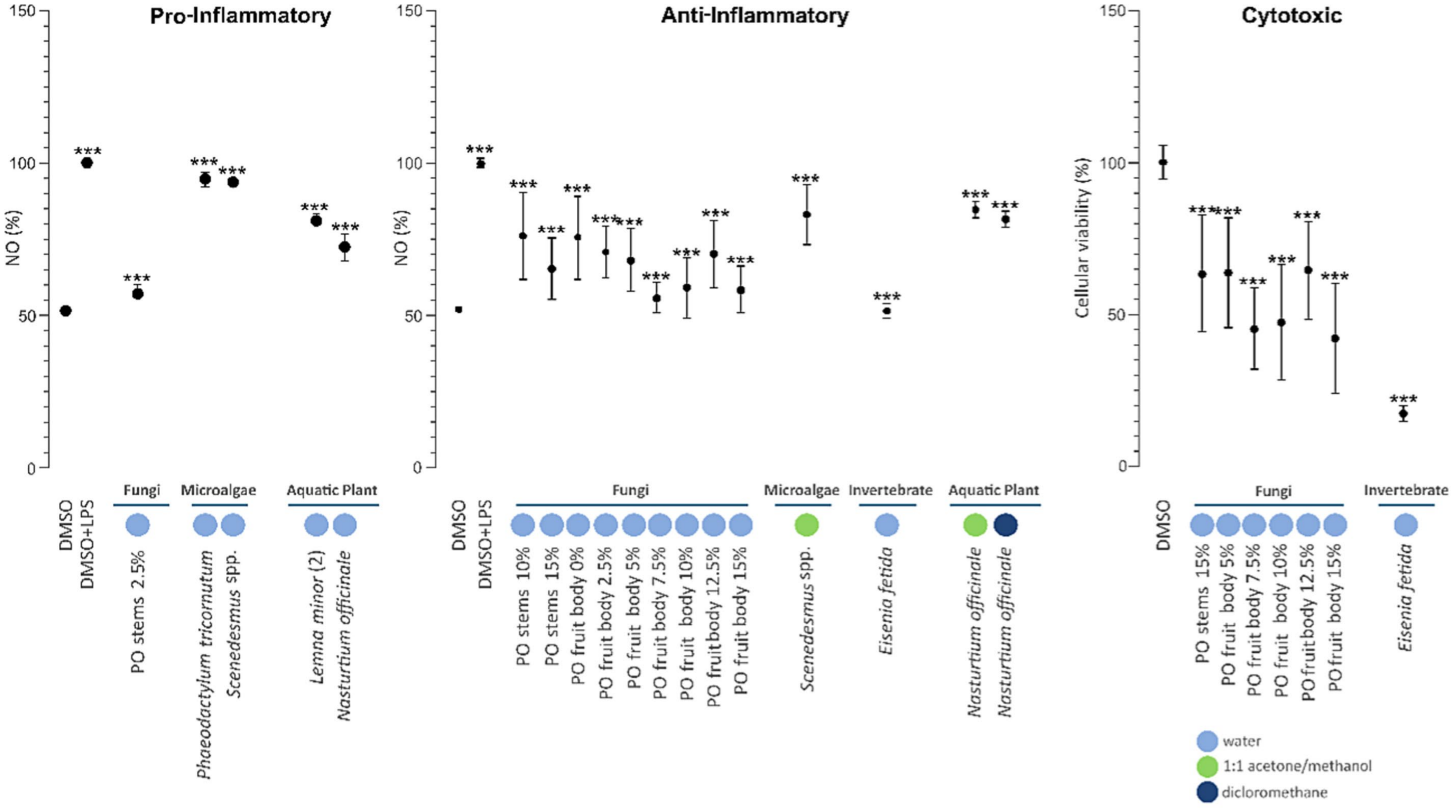
Extract amount (mg) obtained from each biomass using three solvent systems: water (light blue), acetone:methanol (1:1 v/v, dark blue), and dichloromethane (green). Data represent cumulative extraction yields per solvent system for each biomass type.

The use of the different extraction solvents aimed to maximize the recovery of structurally and chemically diverse metabolites and comparatively assess the extraction efficiency of each solvent system across the various biomass types. Extraction *recovered amount* varied between solvents and biomass types, reflecting differences in solvent polarity, solubility of target compounds, and biochemical composition of each biomass (Figure 1). Water consistently produced the highest extraction amounts across all biomass types, underscoring its efficacy in recovering polar, water-soluble compounds such as peptides, amino acids, carbohydrates, and hydrophilic secondary metabolites. Acetone/methanol (1:1, v/v) solvent system provided intermediate amounts and proved particularly effective in extracting both polar and semi-polar metabolites likely including polyphenols, alkaloids, and certain lipid classes. In contrast, dichloromethane yielded the lowest extract quantities overall, consistent with its narrow selectivity for non-polar constituents such as lipids, sterols, and terpenoids, which should represent only a subset of the metabolites present in the analysed biological materials. The highest overall extraction amount was obtained from *P. ostreatus* fruit body cultivated on 10% (w/w) *T. molitor* frass, yielding 1747.0 mg of crude extract (34.9% DW) – predominantly from the aqueous phase (1463.8 mg; 29.3% DW), with smaller contributions from acetone/methanol (229.9 mg; 4.6% DW) and dichloromethane (53.3 mg; 1.1% DW). These values fall within the range reported for aqueous extracts of *P. ostreatus*, which have been documented at 20–40% DW, depending on temperature and extraction conditions (Afonso et al., 2025). Conversely, the lowest amount was recorded for *Lemna minor* (Macusi et al., 2023), with a total of 141.5 mg (2.8% DW) extracted; the majority of this (128.6 mg; 2.6% DW) came from water, and minimal amounts were obtained from the organic solvents. Published solvent-screening studies on *L. minor* generally report higher recoveries: 18.4% DW for ethanol extracts, 17.6% DW with methanol, 13.2% DW for aqueous extracts, 1.43% with chloroform, and 1.35% with hexane from dried *L. minor* (González-Renteria et al., 2020). These data place our recovered amounts at the lower end for water and far below typical alcohol-based extractions, while remaining consistent with the very low recoveries expected for non-polar solvents. The overall pronounced differences observed in our work highlight the strong influence of both biomass type and solvent system on extraction efficiency, reflecting variation in metabolite composition and polarity. Our data also support the relevance of using a multi-solvent extraction approach to maximize chemical diversity recovery and to reveal biomass-specific extraction profiles relevant for downstream bioactivity screening and bioprospecting efforts.

### 3.3 Bioactivity profiling of the biomasses

Following extraction, all crude extracts were subjected to a broad range of *in vitro* bioactivity assays, including prebiotic, antimicrobial, antioxidant, pro- and anti-inflammatory, and cytotoxic assays, designed to evaluate key functional properties relevant to the potential development of novel feed additives with health-modulating properties in aquaculture systems. All extracts were tested at standardized concentrations, and bioactivity responses were quantified relative to appropriate controls, providing an initial broad-range screening of their biotechnological potential.

#### 3.3.1 Prebiotics

**Figure 2:**
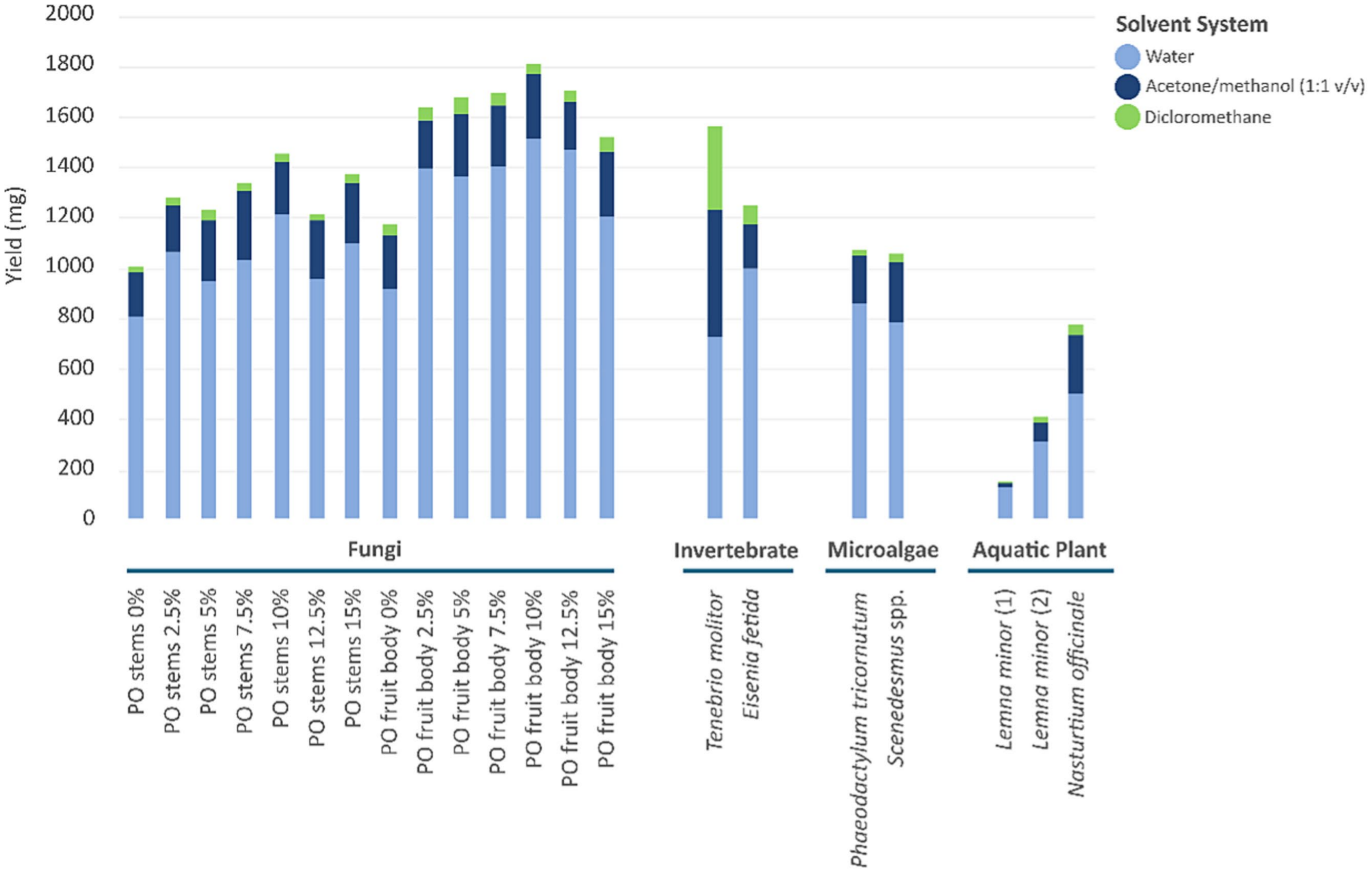
Growth curves of tested probiotic bacterial strains in response to biomass supplementation over 96 h. Each column represents a biomass group: fungi (grey), invertebrates (pink), microalgae (green), and aquatic plants (blue). Each line represents the response of a probiotic strain. Black lines in each panel represent bacterial growth in unsupplemented BMS.

All strains, except *B. longum* ATCC 15708, exhibited growth in at least one biomass-enriched substrate, both in liquid and solid media, while growth in unsupplemented BMS was negligible, confirming that the biomass provided the sole carbon and nutrient sources (Figure 2; Supplementary Table S4). *B. spizizenii* ATCC 6633 showed consistent growth in response to nearly all biomass substrates. The highest OD_600_ values at 96 h were observed with *P. ostreatus* fruit bodies supplemented with 12.5% frass (1.688), followed by 5% (1.655) and 10% (1.469). *Scenedesmus* spp. (OD_600_ = 1.322) also strongly supported bacterial proliferation. Among all tested strains, *P. chlororaphis* subsp*. piscium* DSM 21509 showed the strongest and most consistent growth across all tested prebiotic strains. At 96 h, five biomass samples – *P. ostreatus* fruit body enriched with 5 to 15% frass – supported maximum bacterial growth of this strain (OD_600_ = 3.000). Other high-performing substrates with top-ranking probiotic strains growth promoters included *T. molitor* (OD_600_ = 2.555) and *Scenedesmus* spp. (OD_600_ = 1.863). For all other biomasses, final OD_600_ values still ranged from 0.430 to 1.636, demonstrating the strain’s broad metabolic versatility and its ability to utilize a wide spectrum of biomass-derived compounds for growth. *L. raffinolactis* SAFE_10 exhibited limited growth across all tested substrates, with OD_600_ values never exceeding 0.5 at 96 h, indicating that the tested substrates showed low prebiotic responsiveness for this strain under the tested conditions. *B. subtilis* ATCC 6051 showed moderate to strong growth across several biomass substrates, with varying patterns over time. The highest OD_600_ at 96 h was observed with *P. ostreatus* fruit body enriched with 15% frass (OD = 1.544), followed by 12.5% (1.455) and 12% (1.412). *P. tricornutum* also induced a strong and progressive growth response, yielding an OD_600_ of 1.333, the same as that obtained with *P. ostreatus* fruit body enriched with 5% frass. The invertebrate-derived biomasses, *T. molitor* and *E. fetida,* yielded similar OD_600_ values at 96 h (1.255 and 1.166, respectively). *L. rhamnosus* ATCC BAA-3227 showed moderate and substrate-dependent growth. At 96 h, the best-performing substrate was *P. ostreatus* stems enriched with 15% frass, which supported the highest growth (OD_600_ = 1.355), followed by 10% (1.220) and 7.5% (1.163). Lower growth was observed in response to invertebrate, microalgae, and plant biomasses, with OD_600_ values never exceeding 0.25 at 96 h. This strain therefore displayed a selective substrate preference, responding best to fungal stem-derived biomasses enriched with *T. molitor* frass, while showing negligible utilization of other biomass types. *L. acidophilus* ATCC 314 displayed limited growth across most biomass substrates, with a few notable exceptions. The highest OD_600_ at 96 h was observed with *E. fetida* (1.032), followed by *Scenedesmus* spp. (0.989) and *T. molitor* (0.745). With all the other biomasses, OD_600_ values remained at or near baseline throughout the incubation period (Figure 2). These results indicate a narrow substrate preference, responding specifically to invertebrate- and microalgae-based biomasses. In summary, the evaluated biomasses demonstrated distinct and selective prebiotic effects on a diverse range of probiotic strains relevant to both fish and human health. While most strains showed enhanced growth with at least one biomass substrate, the degree and specificity of growth varied notably among strains. *P. chlororaphis* subsp*. piscium* DSM 21509 exhibited the broadest metabolic versatility, thriving on nearly all biomass types, particularly *P. ostreatus* enriched with *T. molitor* frass. In contrast, strains like *L. raffinolactis* SAFE_10 and *L. acidophilus* ATCC 314 showed limited responsiveness, reflecting narrower substrate preferences. Prior studies on the same species tested in our work have reported prebiotic-like properties or the ability to modulate beneficial microbes: mushroom polysaccharides, especially *β*-glucans from *P. ostreatus*, have been shown to enhance the growth of lactic acid bacteria and bifidobacteria *in vitro* and to modulate gut communities *in vivo* (Yu et al., 2023); microalgae/diatoms such as *P. tricornutum* can shift microbiota composition and increase short-chain fatty acids in animals (Stiefvatter et al., 2022), and microalgal extracts can stimulate probiotic growth and confer protective effects *in vitro* (Carballo et al., 2019); invertebrate-derived materials, such as *T. molitor* and *E. fetida* contain chitin and chitosan, which consistently exhibit prebiotic activity for *Lactobacillaceae* and *Bifidobacteriaceae* (Lopez-Santamarina et al., 2020; Zacharis et al., 2024); duckweeds (*Lemna* spp.) can serve as substrates for probiotic *Bacillus* propagation and have been explored as fermentable feeds (Mahoney et al., 2021). Overall, fungal biomasses, invertebrate- and microalgae-derived substrates showed the most consistent prebiotic-like stimulation, highlighting their potential for targeted probiotic support and biotechnological applications.

#### 3.3.2 Antimicrobials

**Figure 3:**
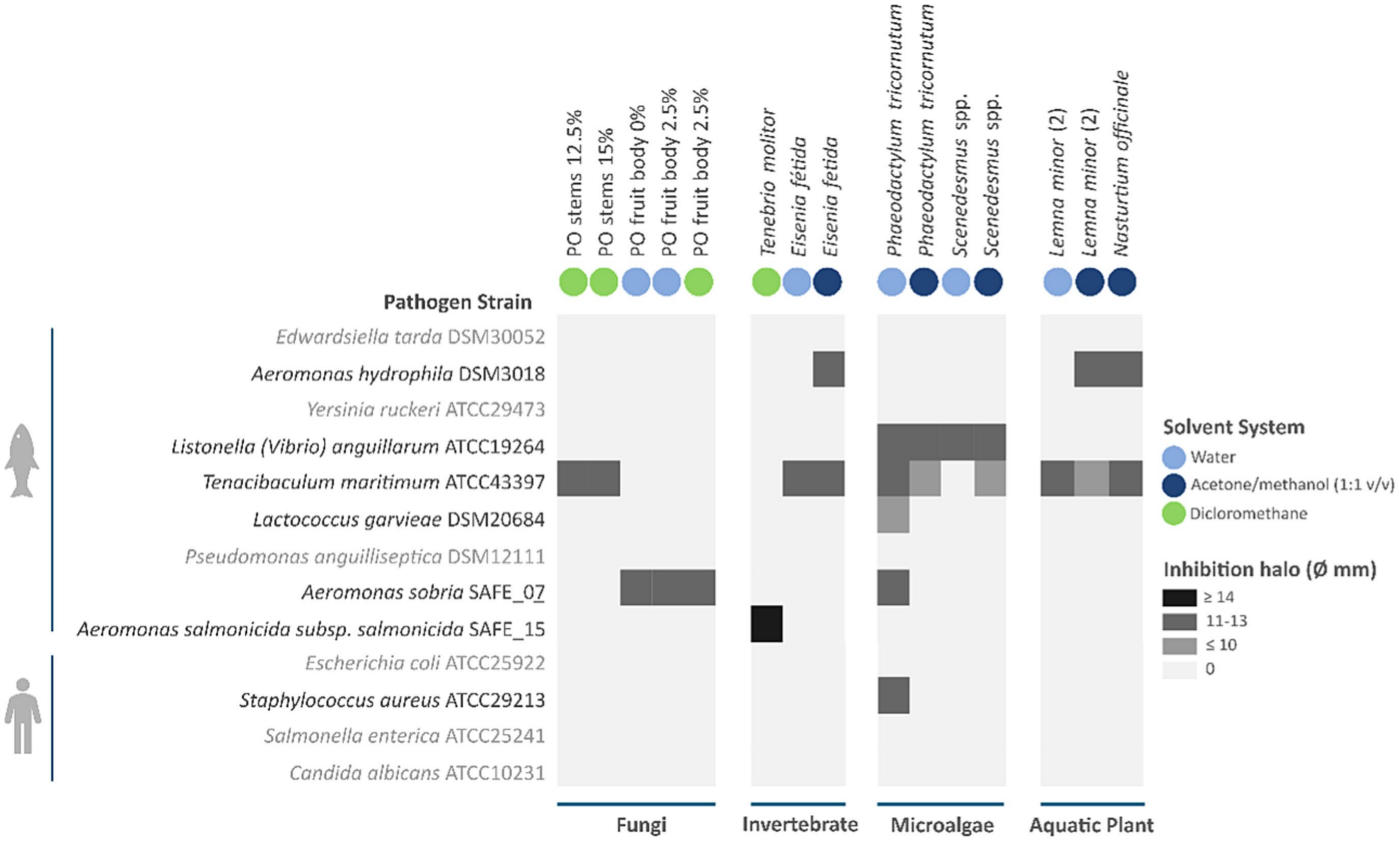
Antimicrobial activity of biomass-derived extracts against a panel of fish and human bacterial pathogens. The solvent system used to obtain each active extract is indicated. Each cell represents the growth inhibition for a given extract–strain combination, based on the diameter of the inhibition halos in the disc diffusion assay. Only extracts exhibiting activity against at least one of the tested pathogens are shown. Data relative to PO was extracted from our previous publication (Hilali et al., 2025).

The antimicrobial activity of the library of crude extracts generated from the biomasses was assessed against a panel of fish and human bacterial pathogens using the disc diffusion assay. Growth inhibition zones were recorded for each extract-strain combination (Figure 3). Of the 63 extracts screened, 20.6% (*n* = 13) exhibited activity against at least one pathogen, with inhibition zones ranging from 9 to 14 mm in diameter. The solvent control (DMSO) did not affect the proliferation of any bacterial strain tested, confirming that the observed inhibitory effects were attributable solely to biomass-derived compounds. Overall, extracts demonstrated greater efficacy against fish-associated pathogens, with measurable inhibition observed in six of the nine tested strains. In contrast, activity against human-associated pathogens was limited, with inhibition detected only for *S. aureus* ATCC 29213 exposed to the aqueous *P. tricornutum* extract. Differences in efficacy were observed among biomass types: microalgae extracts were the most active, inhibiting five distinct pathogens, followed by invertebrate-derived extracts (which inhibited three pathogens), and fungal and aquatic plant extracts (inhibiting two pathogens). Pathogen susceptibility varied, with *T. maritimum* ATCC 43397 being the most sensitive, followed by *L. anguillarum* ATCC 19264 and *A. sobria* SAFE_07, each inhibited by multiple biomass-solvent combinations. Notably, all active microalgal extracts inhibited *L. anguillarum*, whereas no other biomass demonstrated activity against this strain. The observed differences between fish- and human-associated pathogens suggest a degree of target specificity. Furthermore, solvent-dependent activity patterns highlight the importance of employing a broad range of solvents to recover both polar and non-polar bioactive compounds. Most of the extracts exhibiting antimicrobial activity were obtained using dichloromethane, followed by the aqueous extracts, and then those recovered using a 1:1 acetone/methanol mixture. These results align with previously reported antimicrobial properties of the tested biomass types. Fungal extracts, particularly from *P. ostreatus*, have been shown to inhibit both Gram-positive and Gram-negative bacteria (Gashaw et al., 2020; Yakobi et al., 2023). Although most studies to date have focused on human clinically relevant pathogens, our recent publication demonstrated for the first time that extracts from *P. ostreatus* partially cultivated on mealworm frass exhibit antimicrobial activity against *T. maritimum* (Hilali et al., 2025), a major marine aquaculture pathogen, supporting their use as sustainable functional ingredients for fish health management. Invertebrate-derived extracts, specifically those from *T. molitor*, rich in chitin and chitosan, and antimicrobial enzymes such as lysozymes and peptides, have demonstrated efficacy against a wide range of *Aeromonas, Vibrio, Pseudomonas*, and *Staphylococcus* species (Ansari and Sitaram, 2011; Hwang et al., 2022; Liu et al., 2004). To our knowledge, this is the first report of *E. fetida* derived extracts displaying inhibitory activity against *T. maritimum*. Microalgae are recognized as prolific producers of a wide range of bioactive metabolites. Among these, *Scenedesmus* spp. (Dantas et al., 2019; Zaharieva et al., 2022) and *P. tricornutum* (Desbois et al., 2008; Desbois et al., 2009) have been reported to yield extracts with antibacterial activity against relevant pathogens, including multidrug-resistant strains. In this work, we expand the repertoire of susceptible microbes by demonstrating inhibition of *T. maritimum*, *A. sobria*, *L. garviae*, and *L. anguillarum,* all major bacterial pathogens in aquaculture. Aquatic plants, such as *L. minor*, have been recognized for their antimicrobial potential. Early studies demonstrated broad-spectrum antibacterial activity of duckweed extracts against human-associated pathogens, including *Staphylococcus*, *Bacillus*, *Citrobacter*, and *Neisseria* species (Gülçin et al., 2010). Later work confirmed activity against *Pseudomonas fluorescens* and highlighted efficacy across various solvent extracts (González-Renteria et al., 2020). However, data targeting aquaculture pathogens remain sparse. In our study, we demonstrate for the first time that *L. minor* extracts inhibit *A. hydrophila* and *T. maritimum*, two critical fish pathogens, thereby expanding the known antimicrobial spectrum of duckweed and strengthening its promise as a sustainable agent for managing bacterial diseases in aquaculture systems. Altogether, our findings identify specific biomass–solvent combinations as promising candidates for the development of functional aquaculture feed ingredients, particularly relevant in production settings where effective control of bacterial pathogens is critical.

#### 3.3.3 Antioxidants

The antioxidant activity of the biomass derived aqueous and acetone/methanol (1:1) extracts was evaluated by their capacity to scavenge DPPH (2,2′-diphenyl-1-picrylhydrazyl) free radicals (Table 2). The extracts obtained with dichloromethane were excluded from the assay due to their poor solubility in methanol. Values ranged from less than 1 μmol TE g^−1^ to more than 13 μmol TE g^−1^ depending on the biomass type and extraction solvent. Aquatic plants, duckweed and watercress, showed the highest activities in aqueous extracts (13.4 and 8.7 μmol TE g^−1^, respectively). These results may indicate a high content of hydrophilic antioxidants such as phenolic compounds, flavonoids, and vitamin C analogues (Martínez-Sánchez et al., 2008; Muller et al., 2025; Takács et al., 2025), which are known to enhance systemic antioxidant capacity in fish (Bešlo et al., 2023). Microalgae exhibited a contrasting antioxidant activity profile, where aqueous *P. tricornutum* extracts showed reduced activity (0.6 μmol TE g^−1^) but the respective acetone/methanol extract showed comparatively higher activity (6.0 μmol TE g^−1^), which may be due to its carotenoid content, such as fucoxanthin (Neumann et al., 2019). These lipophilic compounds are readily absorbed and deposited in fish tissues, where they can act as chain-breaking antioxidants and protect cellular components from oxidative damage (Xiao et al., 2020). Fungal biomass, particularly aqueous extracts of *P. ostreatus* fruiting body, showed low antioxidant activity (1.6–3.2 μmol TE g^−1^), while the aqueous extracts of stems and acetone/methanol extracts of both fruit bodies and stems showed a consistently lower antioxidant capacity. These results indicate a low abundance of hydrophilic compounds, which could contribute to the free radical scavenging capacity when included in aquafeeds. In the same regard, the studied invertebrate extracts showed the lowest values (around 1–2 μmol TE g^−1^), suggesting that while they represent a sustainable protein source, their role as a source of antioxidant compounds may be limited. Altogether, these results suggest that biomass sources differ not only in the magnitude of antioxidant activity but also in the type of compounds they provide. Aquatic plants showed a significantly higher antioxidant capacity, whereas microalgae showed more moderate antioxidant activity, depending on the polar nature of the extracted compounds. Given the negative impact of oxidative stress on fish growth, immunity, and overall welfare, incorporating antioxidant-rich biomasses as functional ingredients into aquafeeds could reduce reliance on synthetic supplements and enhance fish resilience (Hu et al., 2025).

**Table 2:** Antioxidant activity of the aqueous and acetone/methanol extracts of each biomass.

Biomass		Organic extraction solvent	
		Water	Acetone/methanol (1:1)
DPPH (μmol TE g^−1^)			
Fungi	P.O. stems 0%	2.73 ± 0.26^cd^	0,26 ± 0.08^A^
	P.O. stems 2.5%	1.25 ± 0.09^abc^	0,46 ± 0.02^A^
	P.O. stems 5%	0.93 ± 0.10^ab^	0,28 ± 0.01^A^
	P.O. stems 7.5%	1.09 ± 0.03^abc^	0,37 ± 0.01^A^
	P.O. stems 10%	1.07 ± 0.09^abc^	0,47 ± 0.01^A^
	P.O. stems 12.5%	0.99 ± 0.03^ab^	0,43 ± 0.01^A^
	P.O. stems 15%	1.85 ± 0.80^abcd^	0,74 ± 0.04^A^
	P.O. fruit body 0%	1.70 ± 0.18^abcd^	0,82 ± 0.01^A^
	P.O. fruit body 2.5%	1.63 ± 0.33^abcd^	0,74 ± 0.08^A^
	P.O. fruit body 5%	2.38 ± 0.49^bcd^	0,58 ± 0.04^A^
	P.O. fruit body 7.5%	2.10 ± 0.03^abcd^	1,15 ± 0.07^AB^
	P.O. fruit body 10%	3.23 ± 0.60^d^	0,96 ± 0.02^A^
	P.O. fruit body 12.5%	2.10 ± 0.10^abcd^	1,30 ± 0.06^AB^
	P.O. fruit body 15%	3.16 ± 0.09^d^	0,90 ± 0.12^A^
Invertebrates	*Tenebrio molitor*	1.76 ± 0.85^abcd^	0.27 ± 0.01^A^
	*Eisenia fetida*	1.05 ± 0.11^abc^	0.31 ± 0.01^A^
Microalgae	*Phaeodactylum tricornutum*	0.60 ± 0.05^a^	6.04 ± 1.34^E^
	*Scenedesmus* spp.	0.85 ± 0.26^ab^	1.33 ± 0.17^AB^
Aquatic Plants	*Lemna minor* (1)	1.66 ± 0.07^abcd^	0.83 ± 0.01^A^
	*Lemna minor* (2)	13.4 ± 0.26^f^	4.51 ± 0.31^D^
	*Nasturtium officinale*	8.71 ± 0.45^e^	2.26 ± 0.02^C^
One-way ANOVA	*p-*value	< 0.001	< 0.001

P.O.: Pleurotus ostreatus. Values are presented (*n* = 2) as mean ± standard deviation. One-way ANOVA: lowercase letters indicate significant differences (*p*-value < 0.05) between the aqueous biomass extracts, while uppercase letters indicate significant differences (*p*-value < 0.05) between the acetone/methanol (1:1) biomass extracts.

#### 3.3.4 Pro- and anti-inflammatory

**Figure 4:**
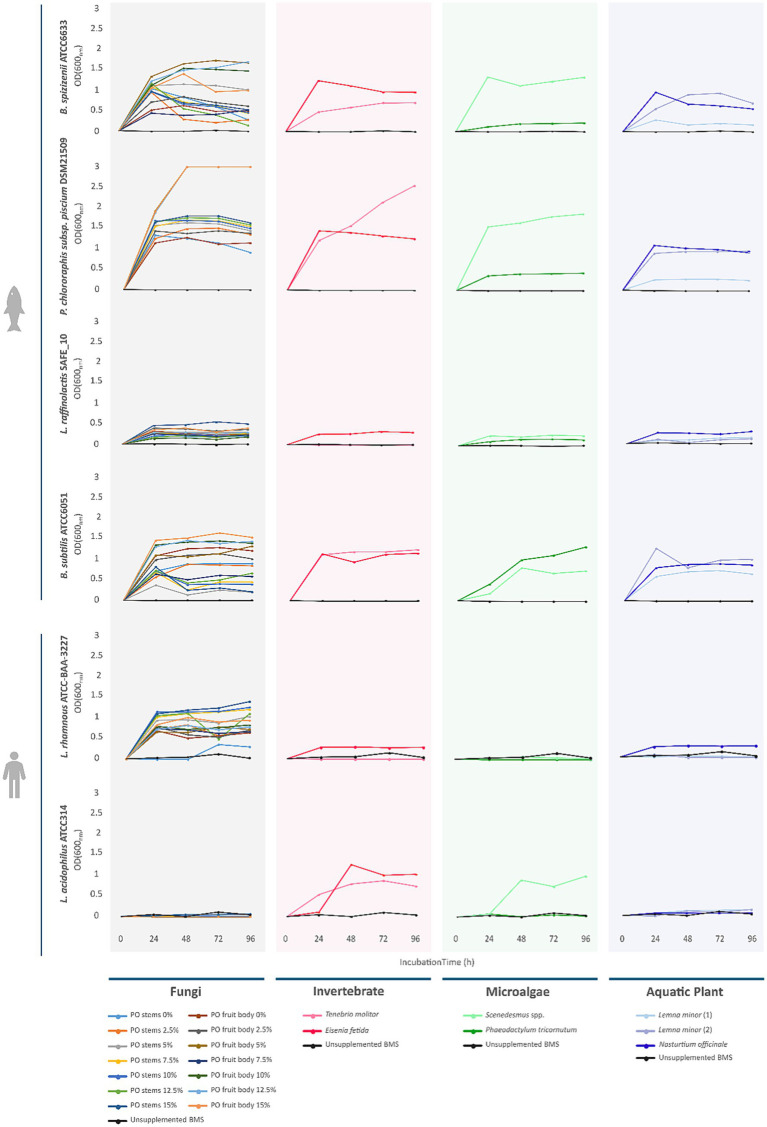
Pro- and anti-inflammatory, and cytotoxic activities of biomass-derived extracts. Pro- and anti-inflammatory activities were measured in RAW 264.7 macrophages as NO production relative to the solvent control (DMSO) or the LPS-stimulated control (DMSO + LPS), respectively. Cytotoxicity was assessed by the MTT assay, expressed relative to the solvent control. The solvent system used to obtain each active extract is indicated. Each data point represents the mean ± standard deviation of two independent biological replicates, each performed in technical triplicate. Only extracts exhibiting statistically significant effects of *p* < 0.001 = *** are shown.

The immunomodulatory potential of the biomass-derived crude extract library was evaluated based on their ability to modulate NO production in RAW 264.7 macrophages, either in the absence (pro-inflammatory assay) or presence (anti-inflammatory assay) of LPS, as well as their impact on cell viability (cytotoxicity assay) (Figure 4). Significant pro-inflammatory activity (*p* < 0.001), measured as an increase in NO production relative to basal levels (solvent control, DMSO), was detected for aqueous extracts from fungi (*P. ostreatus* stems, 2.5%), microalgae (*P. tricornutum* and *Scenedesmus* spp.), and aquatic plants (*L. minor* and *N. officinale*), with increases ranging from 10 to >40%. Given their detrimental potential for aquaculture nutrition, these extracts were removed from the valorization pipeline. Significant anti-inflammatory activity, indicated by reduced NO production in LPS-stimulated cells relative to the DMSO + LPS control, was observed across a broader spectrum of biomass types, including fungal extracts (*P. ostreatus* stems 10–15%, fruit bodies 0–15%), microalgae (*Scenedesmus* spp.), annelid (*E. fetida*), and aquatic plant (*N. officinale*), with reductions between 20 and 50%. Importantly, several extracts with strong anti-inflammatory activity, namely *P. ostreatus* stems (10%), fruit bodies (0–2.5%), *Scenedesmus* spp., and *N. officinale*, did not cause any significant reduction in RAW 264.7 cell viability, demonstrating that their NO-lowering effects were not attributable to cytotoxicity. All the remaining ones were considered as “false positives” and omitted from further consideration. These non-cytotoxic, anti-inflammatory profiles are particularly promising as functional feed ingredients, offering bioactive properties that may help modulate inflammatory responses in aquaculture species without compromising cell health. These results are consistent with previous reports showing that edible mushrooms, including *P. ostreatus*, and microalgae, such as *P. tricornutum*, can modulate macrophage NO production and inflammatory cytokines through pathways including NF-κB and iNOS, without inducing cytotoxicity (Jedinak et al., 2011; Llauradó Maury et al., 2021; Riccio and Lauritano, 2019). In contrast, the invertebrate-derived *E. fetida* extract, despite showing anti-inflammatory activity, markedly reduced macrophage viability (<20%), indicating strong cytotoxic potential and limiting its suitability for feed applications. Taken together, these findings identify *P. ostreatus* fruit bodies 0–2.5% and stems 10%, *Scenedesmus* spp., and *N. officinale* as the most promising biomass sources for the development of novel aquafeed ingredients with targeted anti-inflammatory activity and minimal cytotoxic risk.

## 4 Conclusion

Valorization of underutilized biomasses derived from European freshwater farming systems offer a promising and necessary route to enhance circular economy practices while simultaneously generating highly valuable bioactive ingredients for functional aquafeeds. This study investigated waste-derived biomasses from diverse biological groups—the fungus *Pleurotus ostreatus*, invertebrates like *Tenebrio molitor* and *Eisenia fetida*, microalgae such as *Phaeodactylum tricornutum* and *Scenedesmus* spp., and aquatic plants (*Lemna minor* and *Nasturtium officinale*). A multi-solvent extraction approach (using water, acetone/methanol, and dichloromethane) was successfully employed to maximize the recovery of structurally and chemically diverse metabolites from these biomasses, which were subsequently screened for prebiotic, antimicrobial, antioxidant, and immunomodulatory properties (Table 3).

**Table 3:** Integrated summary of the bioactive properties of biomass-derived crude extracts evaluated in this study.

Biomass		Bioactivity			
		Antimicrobial	Prebiotic	Anti-inflammatory	Anti-oxidant
Fungi	P.O. stems 0%	−	+	−	−
	P.O. stems 2.5%	−	+	−	−
	P.O. stems 5%	−	+	−	−
	P.O. stems 7.5%	−	+	−	−
	P.O. stems 10%	−	+	+	−
	P.O. stems 12.5%	+	+	−	−
	P.O. stems 15%	+	+	−	−
	P.O. fruit body 0%	+	+	+	−
	P.O. fruit body 2.5%	+	+	+	−
	P.O. fruit body 5%	−	+	−	−
	P.O. fruit body 7.5%	−	+	−	−
	P.O. fruit body 10%	−	+	−	−
	P.O. fruit body 12.5%	−	+	−	−
	P.O. fruit body 15%	−	+	−	−
Invertebrates	*Tenebrio molitor*	+	+	−	−
	*Eisenia fetida*	+	+	−	−
Microalgae	*Phaeodactylum tricornutum*	+	+	−	−
	*Scenedesmus* spp.	+	+	+	−
Aquatic plants	*Lemna minor* (1)	−	+	−	−
	*Lemna minor* (2)	+	+	−	+
	*Nasturtium officinale*	+	+	+	+

The biomasses are classified as active (+) or non-active (−) based on the criteria described in the prior section.

The screening yielded several key findings. In terms of prebiotic activity, the biomasses exhibited distinct and selective effects, enhancing the growth of all tested probiotic strains—except *Bifidobacterium longum* ATCC15708, with at least one substrate. Notably, the fish-relevant probiotic *P. chlororaphis* subsp*. piscium* DSM 21509 demonstrated the broadest metabolic versatility, achieving maximum growth (OD_600_ = 3.000), when supplemented with *P. ostreatus* fruit body enriched with *T. molitor* frass. Furthermore, significant antimicrobial activity was observed, with extracts from fungi, invertebrates, microalgae, and aquatic plants (*P. ostreatus*, *T. molitor*, *E. fetida*, *L. minor*) proving effective activity against several major fish pathogens, including *A. hydrophila*, *A. sobria*, *A. salmonicida*, *L. garvieae*, *L. anguillarum*, and *T. maritimum*. Among these, the microalgae extracts were the most active group, inhibiting five distinct pathogens. Several biomasses (fungal, microalgal, and aquatic plant) extracts also exhibited significant anti-inflammatory activity (immunomodulatory potential) in RAW 264.7 macrophage cells by reducing nitric oxide (NO) production. This benefit was achieved without negatively affecting cell viability. Lastly, when assessing antioxidant capacity, aqueous extracts of aquatic plants displayed the highest DPPH radical scavenging capacity, whereas the other biomasses exhibited comparatively low antioxidant potential. Interestingly, *N. officinale* was the only biomass which crude extracts were effective in all the bioactivity screenings performed.

These findings collectively provide novel data supporting the sustainable use of alternative biomasses, produced under circular economy principles, as promising and cost-effective functional ingredients. The identified bioactive properties are suitable for developing innovative, aquafeed ingredients that can contribute significantly to improve animal health and a necessary reduction in antibiotic dependency with the aquaculture sector.

### 4.1 Future perspectives

Future studies should prioritize the addition of the most promising extracts, especially those showing robust prebiotic and antimicrobial efficacy, into experimental diets to fully characterize their effects on growth performances, immune status, and disease resistance under fish farming conditions. This transition from *in vitro* screening to *in vivo* application requires close collaboration with veterinary and fish health specialists to ensure that laboratory findings translate effectively into biological resilience. Furthermore, the chemical characterization of the most potent extracts is essential to isolate and identify the specific key bioactive molecules (e.g., phenolics, polysaccharides, specific peptides) responsible for the observed activities. Partnering with analytical biochemists and pharmacologists will be vital in this phase to map the molecular pathways of these compounds and establish safe, standardized dosages. This targeted approach will enable the development of standardized, concentrated, and highly effective functional ingredient formulations that can be consistently applied by the aquafeed industry, accelerating the transition towards more sustainable, health-promoting, and antibiotic-free aquaculture practices. Finally, engaging with industrial engineers and regulatory experts is crucial to bridge the gap between small-scale circular economy models and large-scale industrial feasibility, ensuring that these innovative solutions are both economically viable and compliant with international safety standards.

## Data Availability

The data presented in this study are publicly available. The data can be found at: https://www.ncbi.nlm.nih.gov/genbank, accession PX617744-57.
