# Supplementary material for: Advancing aquafeed: prebiotic, antimicrobial, antioxidant, and immunomodulatory benefits of innovative ingredients from farming systems
Source: Front Microbiol. 2026 Feb 20;17:1764214. doi: 10.3389/fmicb.2026.1764214 (PMC12963227; doi:10.3389/fmicb.2026.1764214)
Supplement: Supplementary file 1 [file Supplementary_file_1.docx]

Supplementary Material

**Table S1.** Taxonomic identification of bacterial strains isolated from *Oncorhynchus mykiss.* Information regarding isolation source (tissue) and genomic data, including the GenBank accession number, is presented.

| **Strain** | **Isolation Source** | **Closest taxonomy according to EzBioCloud database** | **16S rRNA gene similarity (%)** | **Sequence length (bp)** | **GenBank accession number** |
| --- | --- | --- | --- | --- | --- |
| SAFE_01 | Epidermal mucus | *Aeromonas sobria* | 100.00 | 1412 | PX617757 |
| SAFE_02 | Epidermal mucus | *Pseudomonas paraglycinae* | 99.93 | 1402 | PX617749 |
| SAFE_06 | Head Kidney | *Pseudomonas vancouverensis* | 99.79 | 1405 | PX617743 |
| SAFE_07** | Head Kidney | *Aeromonas sobria* | 100.00 | 1358 | PX617742 |
| SAFE_08 | Head Kidney | *Aeromonas sobria* | 99.86 | 1388 | PX617741 |
| SAFE_09 | Head Kidney | *Aeromonas allosaccharophila* | 100.00 | 1410 | PX617740 |
| SAFE_10* | Head Kidney | *Lactococcus raffinolactis* | 99.79 | 1426 | PX617756 |
| SAFE_11 | Head Kidney | *Lactococcus raffinolactis* | 99.58 | 1423 | PX617755 |
| SAFE_14 | Brain | *Pseudomonas pharyngis* | 100.00 | 1404 | PX617754 |
| SAFE_15** | Brain | *Aeromonas salmonicida subsp. salmonicida* | 100.00 | 1410 | PX617753 |
| SAFE_16 | Brain | *Staphylococcus edaphicus* | 100.00 | 1423 | PX617752 |
| SAFE_17 | Brain | *Lactococcus raffinolactis* | 99.79 | 1426 | PX617751 |
| SAFE_18 | Brain | *Yersinia intermedia* | 99.72 | 1409 | PX617750 |
| SAFE_20 | Brain | *Acinetobacter tjernbergiae* | 99.00 | 1408 | PX617748 |
| SAFE_21 | Brain | *Staphylococcus pasteuri* | 99.86 | 1425 | PX617747 |
| SAFE_22 | Brain | *Acinetobacter albensis* | 99.72 | 1407 | PX617746 |
| SAFE_24 | Head Kidney | *Iodobacter limnosediminis* | 98.85 | 1398 | PX617745 |
| SAFE_27 | Brain | *Chryseobacterium piscicola* | 99.93 | 1379 | PX617744 |

(*) strain used in the prebiotic assay

(**) strain used in the antimicrobial assay

**Table S2.** Strain-specific culture conditions of probiotics used in the *in vitro* prebiotic screening.

| **Strain** | **Host** | **Culture Medium** | **Incubation Time (h)** | **Incubation Temperature (°C)** | **Oxygen Condition** |
| --- | --- | --- | --- | --- | --- |
| **Bacillus spizizenii** ATCC6633 | Fish | Nutrient broth | 24 | 37 | Aerobic |
| **Bacillus subtilis** ATCC6051 | Fish | Nutrient broth | 24 | 30 | Aerobic |
| **Pseudomonas chlororaphis subsp. piscium** DSM21509 | Fish | Tryptic soy | 24-48 | 28 | Aerobic |
| *Lactococcus* *raffinolactis* SAFE**_**10 | Fish | Tryptic soy | 48-72 | 28 | Facultative Anaerobic |
| **Bifidobacterium longum** ATCC15708 | Human | ATCC Medium: 2107 Modified Reinforced Clostridial | 24-48 | 37 | Anaerobic |
| **Lactobacillus acidophilus** ATCC314 | Human | ATCC Medium: 416 Lactobacilli MRS | 24-48 | 37 | Microaerophilic |
| **Lactobacillus rhamnous** ATCCBAA-3227 | Human | ATCC Medium: 416 Lactobacilli MRS | 24-48 | 37 | Microaerophilic |

**Table S3.** Strain-specific culture conditions of pathogens used in the *in vitro* antimicrobial screening.

| **Strain** | **Host** | **Culture Medium** | **Incubation Time (h)** | **Incubation Temperature (°C)** | **Oxygen Condition** |
| --- | --- | --- | --- | --- | --- |
| *Aeromonas hydrophila* DSM3018 | Fish | Tryptic soy | 24 | 28 | Facultative Anaerobic |
| *Edwardsiella* *tarda* DSM30052 | Fish | Tryptic soy | 24 | 28 | Facultative Anaerobic |
| *Lactococcus* *garvieae* DSM20684 | Fish | Tryptic soy | 24-48 | 28 | Facultative Anaerobic |
| *Listonella* (*Vibrio*) *anguillarum* ATCC19264 | Fish | Tryptic soy | 48 | 28 | Facultative Anaerobic |
| *Pseudomonas* *anguilliseptica* DSM12111 | Fish | Tryptic soy | 48-72 | 25 | Aerobic |
| *Tenacibaculum* *maritimum* ATCC43397 | Fish | Marine broth | 48-72 | 25 | Aerobic |
| *Yersinia* *ruckeri* ATCC29473 | Fish | Tryptic soy | 24-48 | 28 | Facultative Anaerobic |
| *Aeromonas* *salmonicida* *subsp*. *salmonicida* SAFE_15 | Fish | Tryptic soy | 48-72 | 28 | Facultative Anaerobic |
| *Aeromonas sobria* SAFE_07 | Fish | Tryptic soy | 48-72 | 28 | Facultative Anaerobic |
| *Candida* *albicans* ATCC10231 | Human | Sabouraud dextrose | 24 | 37 | Aerobic |
| *Escherichia* *coli* ATCC25922 | Human | Mueller-Hinton | 24 | 37 | Facultative Anaerobic |
| *Salmonella* *enterica* ATCC25241 | Human | Mueller-Hinton | 24 | 37 | Facultative Anaerobic |
| *Staphylococcus* *aureus* ATCC29213 | Human | Mueller-Hinton | 24 | 37 | Facultative Anaerobic |

**Table S4.** Growth performance of probiotic strains on different biomass substrates in solid media assay.

| **Biomass** | | | **Probiotic Strain** | | | | | | |
| --- | --- | --- | --- | --- | --- | --- | --- | --- | --- |
|  |  |  | **Aquaculture-relevant** | | | | **Human-health relevant** | | |
|  |  |  | ***B. spizizenii* ATCC6633** | ***P. chlororaphis subsp. piscium* DSM21509** | ***B. subtilis* ATCC6051** | ***Lactococcus raffinolactis SAFE_10*** | ***B. longum* ATCC15708** | ***L. rhamnosus* ATCCBAA3227** | ***L. acidophilus* ATCC314** |
| **Fungi** |  | PO stems 0% | + | + | ++ | + | - | - | - |
|  |  | PO stems 2.5% | + | ++ | ++ | + | - | - | - |
|  |  | PO stems 5% | ++ | ++ | - | + | - | - | - |
|  |  | PO stems 7.5% | ++ | ++ | + | + | - | - | - |
|  |  | PO stems 10% | ++ | ++ | + | + | - | - | - |
|  |  | PO stems 12.5% | ++ | + | ++ | + | - | + | - |
|  |  | PO stems 15% | ++ | + | - | + | - | + | - |
|  |  | PO fruit body 0% | ++ | + | ++ | + | - | + | - |
|  |  | PO fruit body 2.5% | ++ | ++ | ++ | + | - | + | - |
|  |  | PO fruit body 5% | ++ | + | ++ | + | - | + | - |
|  |  | PO fruit body 7.5% | ++ | ++ | + | + | - | + | - |
|  |  | PO fruit body 10% | ++ | + | ++ | - | - | + | - |
|  |  | PO fruit body 12.5% | ++ | + | ++ | + | - | + | - |
|  |  | PO fruit body 15% | ++ | ++ | ++ | + | - | + | - |
| **Invertebrates** | | *Tenebrio molitor* | ++ | ++ | ++ | - | - | - | ++ |
|  |  | *Eisenia fetida* | ++ | ++ | ++ | + | - | - | ++ |
| **Microalgae** | | *Phaeodactylum tricornutum* | + | + | ++ | - | - | - | - |
|  |  | *Scenedesmus* spp. | ++ | ++ | + | + | - | - | ++ |
| **Aquatic Plants** | | *Lemna minor* (1) | + | + | + | - | - | - | - |
|  |  | *Lemna minor* (2) | ++ | ++ | ++ | - | - | - | - |
|  |  | *Nasturtium officinale* | + | ++ | ++ | + | - | - | - |
| **Negative Control** (unsupplemented BMS) | | | - | - | - | - | - | - | - |

(-) No bacterial growth; (+) Visible bacterial growth; (++) High bacterial growth.
